# Supplementary material for: Light Absorption Enhancement and Laser-Induced Damage Ability Improvement of Aluminum Alloy 6061 with Non-Porous Alumina/CdSe@Al2O3/SiO2 Functional Gradient Films
Source: Nanomaterials (Basel). 2022 Feb 6;12(3):559. doi: 10.3390/nano12030559 (PMC8840421; doi:10.3390/nano12030559)
Supplement: Supplementary file 1 [file nanomaterials-12-00559-s001.zip › nanomaterials-1562411-supplementary.pdf]

nanomaterials

Supplementary Materials

Light Absorption Enhancement and Laser-Induced Damage Ability Improvement of Aluminum Alloy 6061 with Non-Porous Alumina/CdSe@Al_2O_3/SiO_2 Functional Gradient Films

Jiaheng Yin, Lihua Lu, Yaowen Cui *, Yongzhi Cao and Yunlong Du

Center for Precision Engineering, Harbin Institute of Technology, Harbin 150001, China; larryyin@hit.edu.cn (J.Y.); lihual@hit.edu.cn (L.L.); cflying@hit.edu.cn (Y.C.); ylhit@hit.edu.cn (Y.D.)

* Correspondence: yaowen.cui897@gmail.com; Tel.: +86-451-86412924; Fax: +86-451-86415244

The experimental system consists of a single longitudinal mode SAGA-S laser Nd: YAG laser, collimated light source, focusing lens, attenuator, aperture, an EPM2000 energy calorimeter, a sample carrier (two-dimensional adjustable, step-by-step accuracy is 10 um), an optical microscope and a computer. The experimental setup is depicted below.

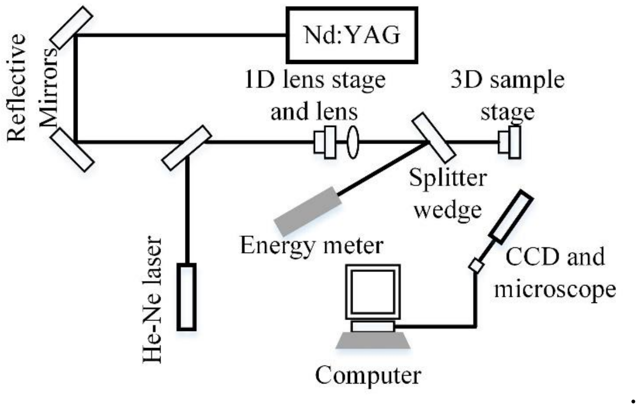

Figure S1. Schematics of laser irradiation damage testing equipment.

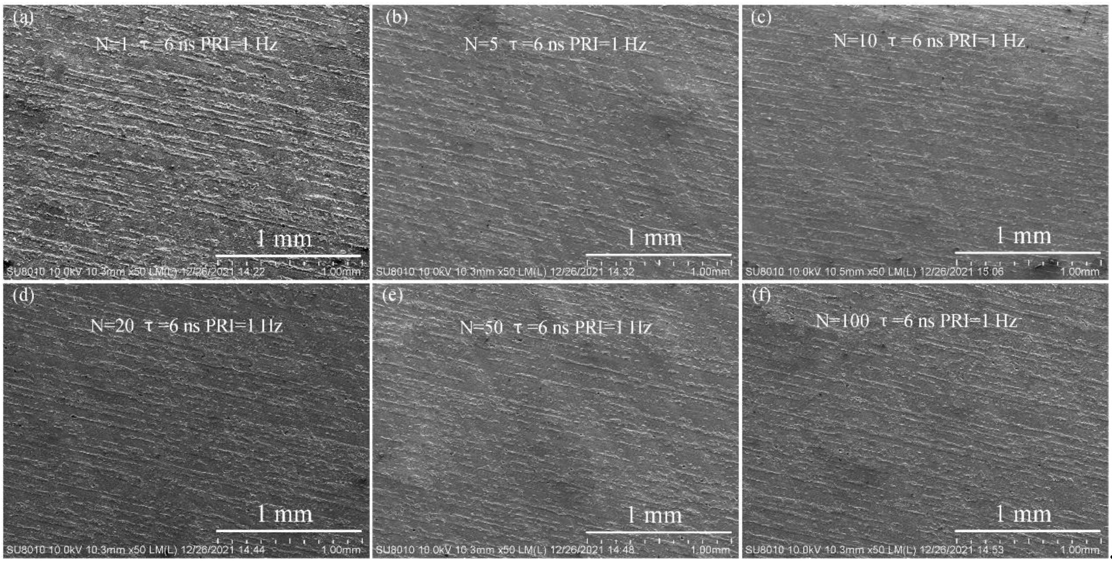

Figure S2. 355-nm Nd:YAG laser irradiation of aluminum alloy 6061 with functional layers at a fluence of 0.5 J/cm^2 at (**a**)1 time, (**b**) 5 time, (**c**) 10 time, (**d**) 20 time, (**e**) 50 time, (**f**) 100 time irradiation.

Table S1. Particles generated by different laser irradiation times with laser fluence of 0.5 J/cm^2 of AA 6061 without functional layers.

S. no	Diameters of particles (µm)				
	**0.3**	**0.5**	**1.0**	**5.0**	**10.0**
1	2141	572	199	3	0
2	10618	4142	1127	25	1
3	1112	252	52	0	0
4	5638	1807	504	6	0
5	1957	538	206	5	0
6	789	144	30	0	0
7	2296	561	145	2	0
8	2514	689	193	9	1
9	2179	459	116	5	0
10	1090	215	62	2	0
11	488	86	13	0	0
12	725	115	18	0	0
13	451	58	5	0	0
14	802	115	20	1	0
15	2182	495	156	9	0

Table S2. Particles generated by different laser irradiation times with laser fluence of 0.5 J/cm^2 with functional layers.

**S. no**	**Diameters of particles (μm)**		
	**0.3**	**0.5**	**1.0**
1	77	16	8
2	380	89	32
3	103	18	13
4	60	21	18
5	87	14	4
6	43	8	4
7	38	1	1
8	37	4	2
9	20	1	0
10	14	1	0
11	21	1	0
12	22	4	1
13	24	1	1
14	21	0	0
15	24	2	2
